# Supplementary material for: Deterministic Fabrication of Plasmonic Nanostructures on Optical Nanofibers via Blurred Electron Beam Deposition
Source: Adv Sci (Weinh). 2025 Jul 11;12(38):e07004. doi: 10.1002/advs.202507004 (PMC12520569; doi:10.1002/advs.202507004)
Supplement: Supplementary file 1 — Supporting Information [file ADVS-12-e07004-s001.docx]

**Deterministic Bottom-Up Fabrication of Plasmonic Nanostructures on Optical Nanofibers via Blurred Electron Beam Deposition**

**SUPPLEMENTARY MATERIAL**

**Antonio Balena^1,*^, Marianna D’Amato^1^, Muhammad Fayyaz Kashif^2,3^, Chengjie Ding^1^, Massimo De Vittorio^3,4^, Ferruccio Pisanello^3^, Alberto Bramati^1^**

***^1^*** *Laboratoire Kastler Brossel, Sorbonne Université, CNRS, ENS-PSL Research University, Collège de France, France*

***^2^*** *Università degli Studi di Napoli Federico II – Department of Electrical Engineering and Information Technologies, Italy*

***^3^*** *Istituto Italiano di Tecnologia - Center for Biomolecular Nanotechnologies, Italy*

***^4^*** *Technical University of Denmark - Department of Health Technology Drug Delivery and Sensing, IDUN Section, Denmark*

***^*^*** *Corresponding author:* [antonio.balena@lkb.upmc.fr](mailto:antonio.balena@lkb.upmc.fr)

# Tapered Optical Nanofiber (TNF) modeling and realization

**Supplementary Figure S1** illustrates the effective refractive index *n_eff_* as a function of the TNF diameter (bottom x-axis) for the first four lower-order modes - *HE_11_*, *HE_21_*, *TM_01_*, and *TE_01_* – considering *n_1_* = 1.46, *n_0_* = 1.00, and *λ* = 785 nm. The effective refractive index *n_eff_* is defined as *n_eff_ = β***/*k*,** where *β* represents the mode propagation constant and *k* = 2*π*/*λ* is the wavenumber. The normalized frequency parameter *V*, is defined as:

$$V=\frac{2\pi}{\lambda}\sqrt{\left( n_{1}^{2}-n_{0}^{2} \right)},$$

and it is displayed on the top x-axis, where *a* is the radius of the TNF*, λ is* the wavelength of interest, and *n_1_* and *n_0_* are the refractive indices of the cladding and surrounding air, respectively*.* In the nanofiber region, as the original core is melted away, the cladding now assumes the role of the core, while air acts as the surrounding medium.

**
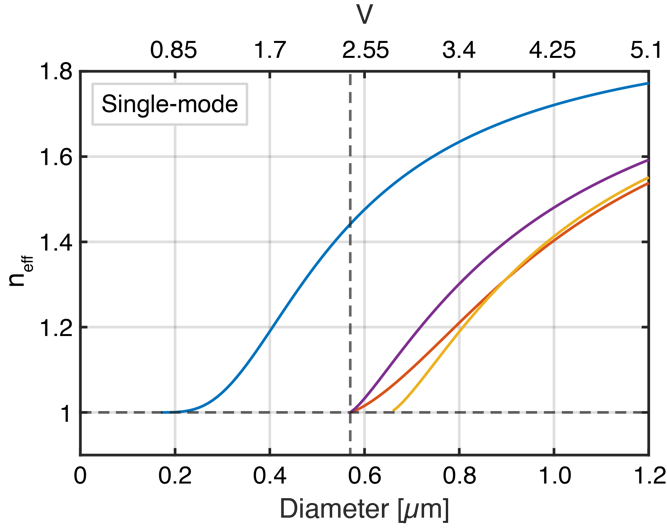
**

Supplementary Figure S1. Effective refractive index n_eff_ as a function of the TNF’s diameter and the normalized frequency parameter V. The boundary between single-mode and multi-mode regimes is marked by a vertical dashed line (V = 2.405). Below this threshold, only the fundamental mode HE11 propagates inside the TNF.

Each mode, except the fundamental *HE_11_* mode, can only propagate if *V* exceeds a mode-specific cut-off value. For *V* < 2.405, only the *HE_11_* mode is supported, meaning the nanofiber operates in a single-mode regime.

## Derivation of the electromagnetic field around the TNF

The fundamental mode *HE_11_* of an TNF is given by the three components of the electric and the magnetic fields *E_x_*, *E_y_*, *E_z_* and *H_x_*, *Hy*, *H_z_* in cylindrical coordinates (*r*, *θ*, *z*). At the air-cladding interface, given the radius *a* of the TNF, these components can be expressed as follows.

For *r* < *a* (inside the fiber):

$$E_{x} = -j A \beta\frac{a}{u}\left[ \frac{\left( 1-s \right)}{2} J_{0}\left( \frac{u}{a}r \right)cos\psi-\frac{\left( 1+s \right)}{2}J_{2} \left( \frac{u}{a}r \right)\cos\left( 2\theta+\psi\right) \right],$$

$$E_{y} = j A \beta\frac{a}{u}\left[ \frac{\left( 1-s \right)}{2} J_{0}\left( \frac{u}{a}r \right)sin\psi+\frac{\left( 1+s \right)}{2}J_{2} \left( \frac{u}{a}r \right)\sin\left( 2\theta+\psi\right) \right],$$

$$E_{y} =A J_{1}\left( \frac{u}{a}r \right) \cos\left( \theta+\psi\right),$$

$$H_{x}=-jA\omega\varepsilon_{0}n_{1}^{2}\frac{a}{u}\left[ \frac{\left( 1-s_{1} \right)}{2}J_{0} \left( \frac{u}{a}r \right)\sin\psi+\frac{\left( 1+s_{1} \right)}{2}J_{2}\left( \frac{u}{a}r \right)\sin\left( 2\theta+\psi\right) \right],$$

$$H_{y}=-jA\omega\varepsilon_{0}n_{1}^{2}\frac{a}{u}\left[ \frac{\left( 1-s_{1} \right)}{2}J_{0} \left( \frac{u}{a}r \right)cos\psi-\frac{\left( 1+s_{1} \right)}{2}J_{2}\left( \frac{u}{a}r \right)\cos\left( 2\theta+\psi\right) \right],$$

$$H_{y}=-A\frac{\beta}{\omega\mu_{0}}s J_{1}\left( \frac{u}{a}r \right)\sin\left( \theta+\psi\right).$$

For *r* > *a* (outside the fiber):

$$E_{x}=-j A \beta\frac{a J_{1}\left( u \right)}{\omega K_{1}\left( \omega\right)}\left[ \frac{\left( 1-s \right)}{2}K_{0}\left( \frac{\omega}{a}r \right)cos\psi+\frac{\left( 1+s \right)}{2}K_{2}\left( \frac{\omega}{a}r \right)\cos\left( 2\theta+\psi\right) \right],$$

$$E_{y}=j A \beta\frac{a J_{1}\left( u \right)}{\omega K_{1}\left( \omega\right)}\left[ \frac{\left( 1-s \right)}{2}K_{0}\left( \frac{\omega}{a}r \right)sin\psi-\frac{\left( 1+s \right)}{2}K_{2}\left( \frac{\omega}{a}r \right)\sin\left( 2\theta+\psi\right) \right],$$

$$E_{z}= A \frac{J_{1}\left( u \right)}{K_{1}\left( \omega\right)}K_{1}\left( \frac{\omega}{a}r \right)\cos\left( \vartheta+\psi\right),$$

$$H_{x}= -jA\omega\varepsilon_{0}n_{0}^{2}\frac{aJ_{1}\left( u \right)}{w K_{1}\left( \omega\right)}\left[ \frac{\left( 1-s_{0} \right)}{2}K_{0} \left( \frac{\omega}{a}r \right)sin\psi-\frac{\left( 1+s_{0} \right)}{2}K_{2}\left( \frac{\omega}{a}r \right)\sin\left( 2\theta+\psi\right) \right],$$

$$H_{y}= -jA\omega\varepsilon_{0}n_{0}^{2}\frac{aJ_{1}\left( u \right)}{w K_{1}\left( \omega\right)}\left[ \frac{\left( 1-s_{0} \right)}{2}K_{0} \left( \frac{\omega}{a}r \right)cos\psi+\frac{\left( 1+s_{0} \right)}{2}K_{2}\left( \frac{\omega}{a}r \right)\cos\left( 2\theta+\psi\right) \right],$$

$$H_{z} = - A\frac{\beta}{\omega\mu_{0}}s \frac{J_{1}\left( u \right)}{K_{1}\left( \omega\right)}K_{1}\left( \frac{\omega}{a}r \right)\sin\left( \theta+\psi\right),$$

Where *ψ* is the polarization angle, ${s = \left[ \left( \omega\right)^{-2} +\left( u \right)^{-2} \right]}/\left[ {J_{1}^{{}^{'}}\left( u \right)}/{{uJ_{1}\left( u \right) + K_{1}^{{}^{'}}\left( \omega\right)}/{\omega K_{1}\left( \omega\right)}} \right]$, ${s_{1}=\beta^{2} s}/\left( k^{2}n_{1}^{2} \right)$, ${s_{0}=\beta^{2} s}/\left( k^{2}n_{2}^{2} \right)$, $\beta$ is the propagation constant, $u = a\sqrt{n_{1}^{2}k^{2}-\beta^{2}}$ and $w = a\sqrt{\beta^{2}-n_{0}^{2}k^{2}}$ ($u^{2}+w^{2}=V^{2}$, where *V* is the normalized frequency). $J_{n}$ and $K_{n}$ are the Bessel functions of the first type and the modified Bessel functions of the second type, respectively. The single quote mark stands for derivative. **Supplementary Figures S2.A-F** show, respectively, the components *E_x_*, *E_y_*, *E_z_*, *H_x_*, *H_y_,* and *H_z_* of the electric and magnetic fields of the fundamental mode *HE_11_* calculated for *λ* = 785 nm guided light linearly polarized along the y-axis for a TNF with a diameter of 360 nm, while **Supplementary Figure S3.A** illustrates the normalized electric field intensity profile (*λ* = 785 nm) for the fundamental mode *HE_11_* as a function of the TNF waist diameter *d*, with light linearly polarized along the horizontal axis (*x* = 0). The center of the TNF is at *y* = 0, and *y* = ±*d*/2 are the upper and lower TNF boundaries. As *d* decreases from right to left, the evanescent field outside the fiber – observed for *y* > *d*/2 or *y* < -*d*/2 – gradually increases, becoming the major contribution, particularly for sub-wavelength *d*. However, when the fiber diameter becomes too small, light confinement to the fiber surface is significantly reduced. The strongest evanescent field at the fiber surface occurs at a fiber waist diameter of *d* = 360 nm, with the corresponding calculated electric field intensity |E|^2^ on the transversal plane shown in **Supplementary** **Figure S3.B** along with detailed equations. A representative SEM Micrograph of a TNF’s minimum waist section is shown in Supplementary **Figure S4.A**, along with an extracted grayscale profile from which it can be observed that the TNF features a waist of *d* = 360 nm, that is maintained along a length of 3 mm. For diameters in the range *d* ϵ [320, 400] nm, the evanescent field intensity at the fiber surface remains above 90% of the maximum (**Supplementary Figure S4.B**), thus defining a tolerance that accounts for slight diameter variations during fabrication.

.


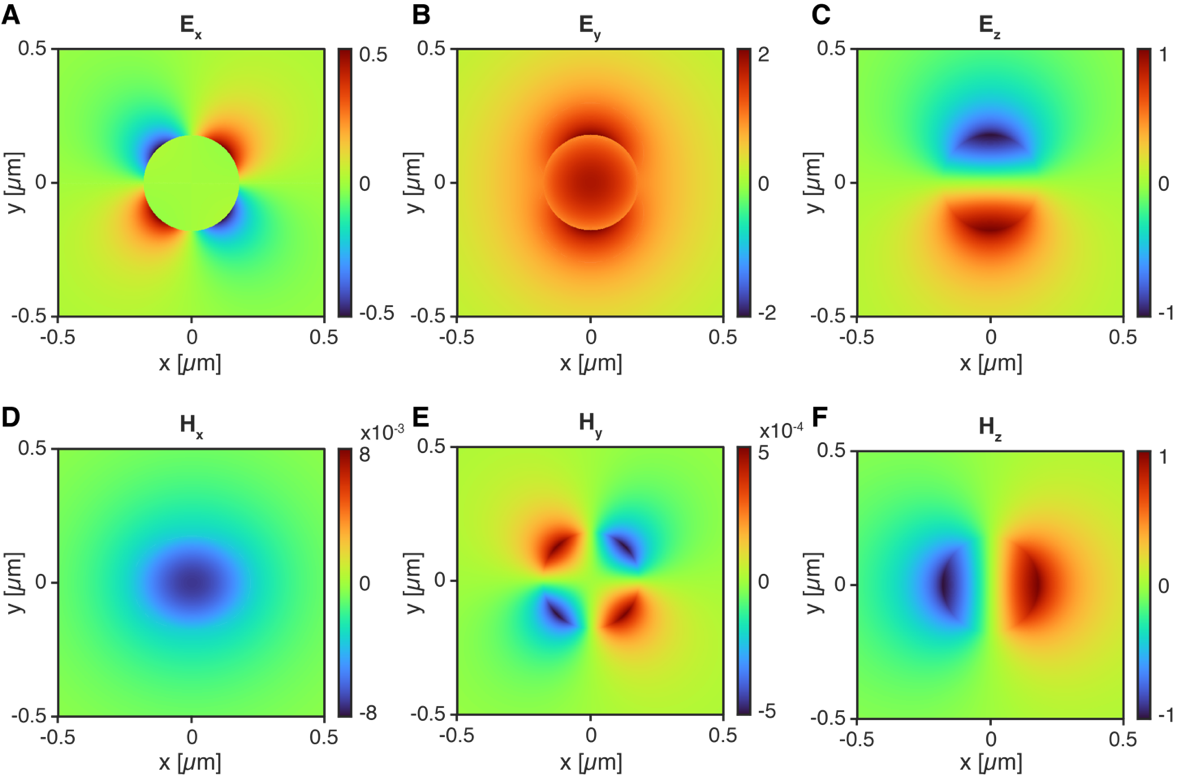


Supplementary Figure S2. Fundamental mode HE_11_ structure. A-F) The components E_x_, E_y_, E_z_, H_x_, H_y,_ and H_z_, respectively, of the electric and magnetic fields of the fundamental mode HE_11_. The components are simulated for a 360 nm-diameter TNF and in the case of a 785 nm guided light with linear polarization along the y-axis using the equations in section S1.1.


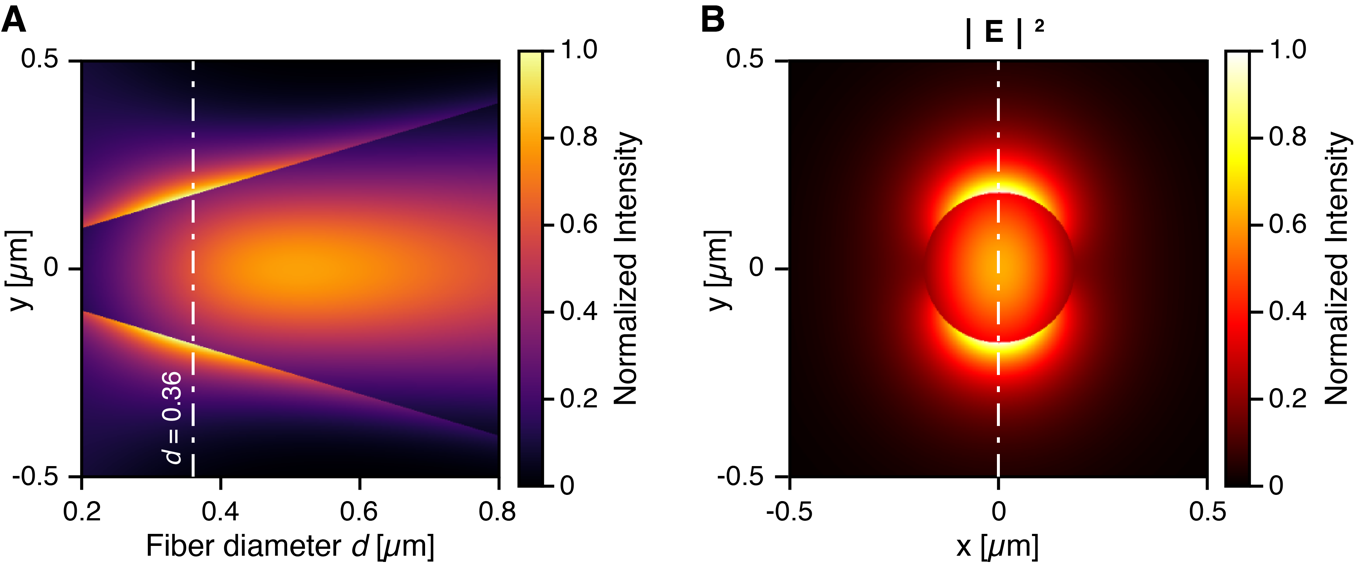


Supplementary Figure S3. electric field intensity calculations. A) Map of the electric field intensity profile along the central axis of the TNF transversal plane versus the TNF diameter for λ = 785 nm. The dot-dashed white line highlights the maximum intensity profile, obtained for a waist diameter of d = 360 nm. B) The normalized electric field intensity distribution on the transversal plane for d = 360 nm and λ = 785 nm.


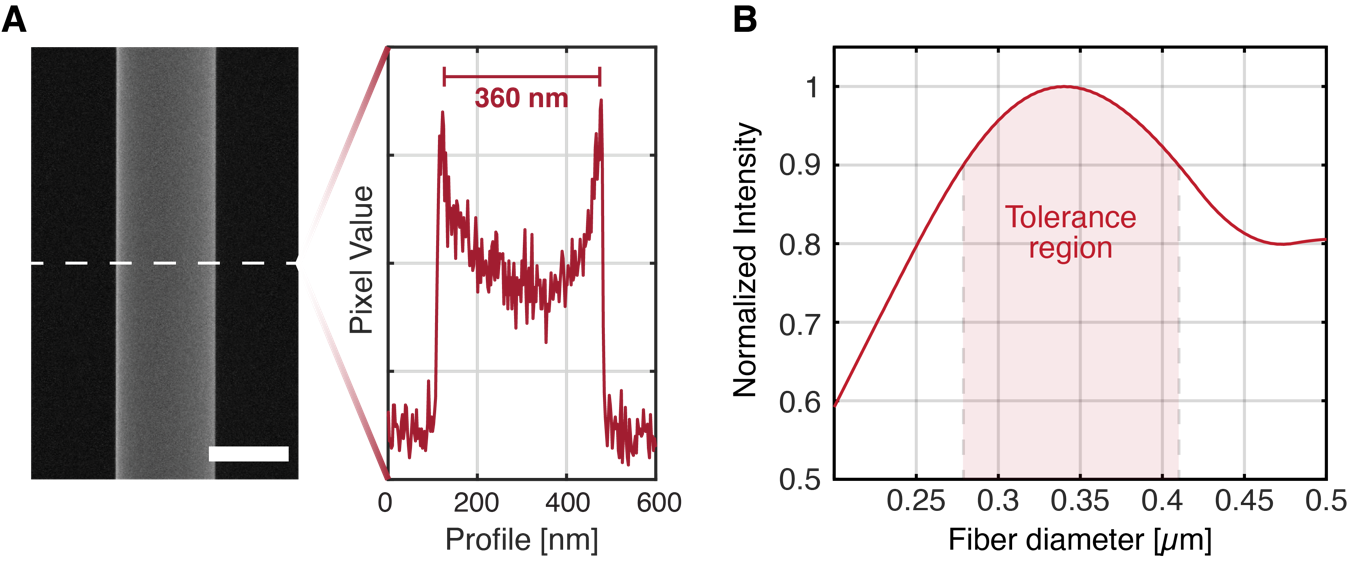


Supplementary Figure S4. Definition of the diameter tolerance region. A) Scanning Electron Micrograph of the minimum waist section of a tapered nanofiber and extracted grayscale values profile along the white dashed line. The scale bar is 300 nm. B) Plot of the maximum electromagnetic field intensity for r > a (evanescent field outside the fiber) versus the nanofiber diameter 2a, normalized at the highest value. The shaded area identifies the range of diameters in which the maximum intensity remains above 90% of the absolute maximum, thereby defining a fabrication tolerance region for the nanofiber.

## TNF’s fabrication and optimization

**
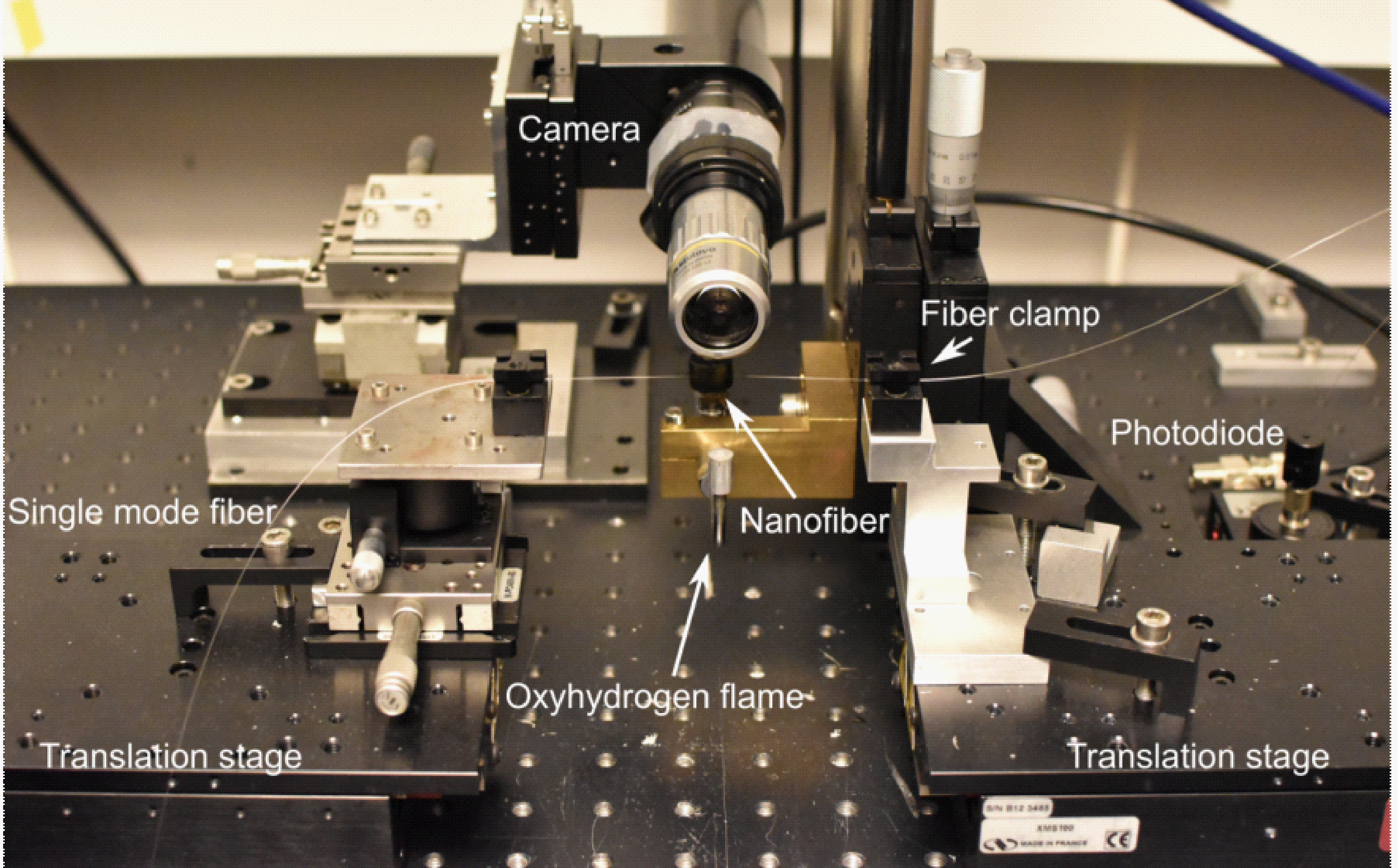
**

Supplementary Figure S5. Custom heat-and-pull setup. The two translation stages follow a precisely calculated pulling trajectory while the oxyhydrogen flame heats the single-mode fiber segment between the two fiber clamps to obtain a nanofiber. A camera continuously monitors the process, and a photodiode retrieves the fiber transmission in real time.


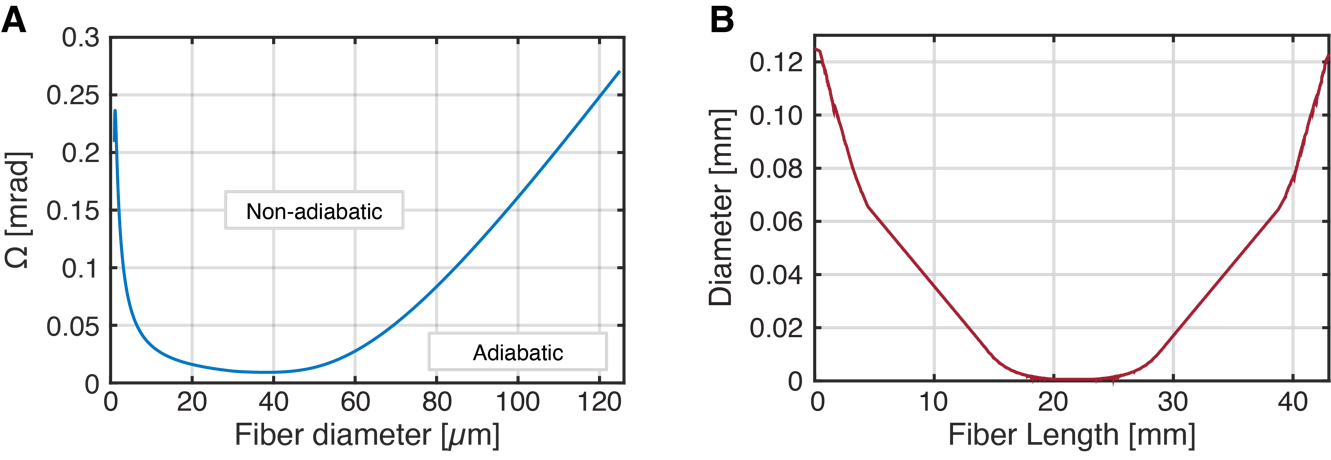


Supplementary Figure S6. Adiabatic condition. A) Adiabatic criterion calculated for a Thorlabs HP780 optical fiber, a diameter d = 360 nm, a nanofiber length of l = 3 mm, and a working wavelength of λ = 785 nm. B) Tapered nanofiber adiabatic profile calculated to obtain adiabatic transmission throughout the entire fiber, satisfying the conditions defined in Panel A.


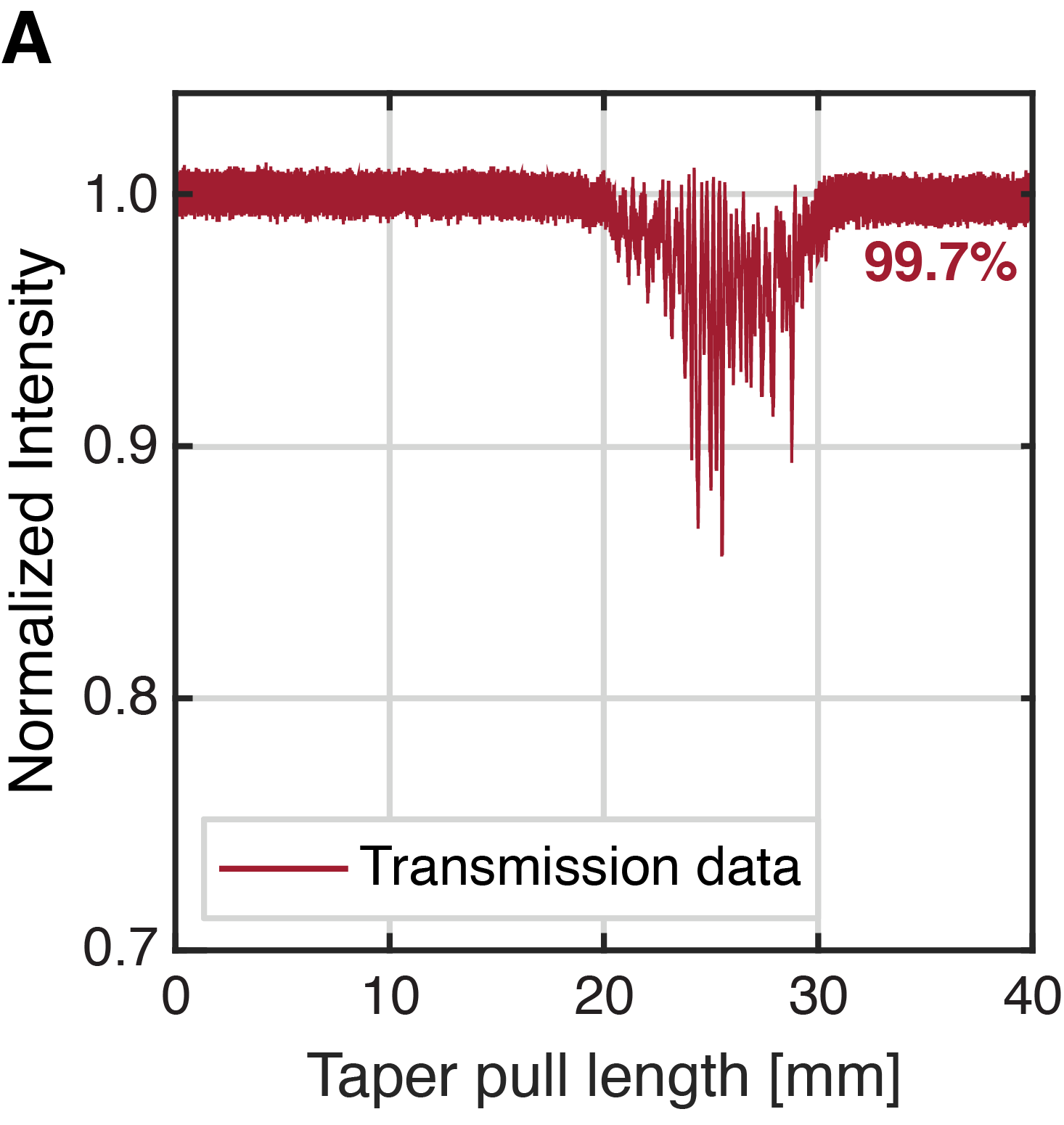


Supplementary Figure S7. Exemplary plot of the measured transmission during the heat-and-pull process, for which the advancement is quantified by the taper pull length. Unitary transmission represents the transmission of the fiber before starting the heat-and-pull.

| **Sample** | **Transmission [%]** | **Waist diameter *d* [nm]** |
| --- | --- | --- |
| Fiber 1 | 99.5 | 380 |
| Fiber 2 | 98.7 | 357 |
| Fiber 3 | 99.5 | 323 |
| Fiber 4 | 99.6 | 364 |
| Fiber 5 | 97.5 | 359 |

Supplementary Table S1. Quality inspection of the fabricated TNFs. Transmission values are relative to the transmission intensity measured before the heat-and-pull process. Waist diameter is measured by Scanning Electron Microscope inspection.


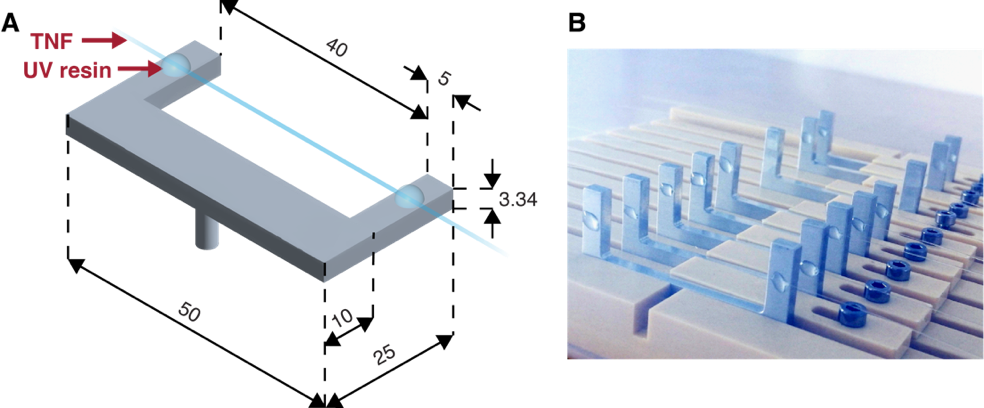


Supplementary Figure S8. Custom holder. A) Sketch of the custom holder designed to insert the TNFs in the SEM machine. TNF not in scale. B) Photograph of prepared nanofibers glued to the custom holder and stored in a clean environment.


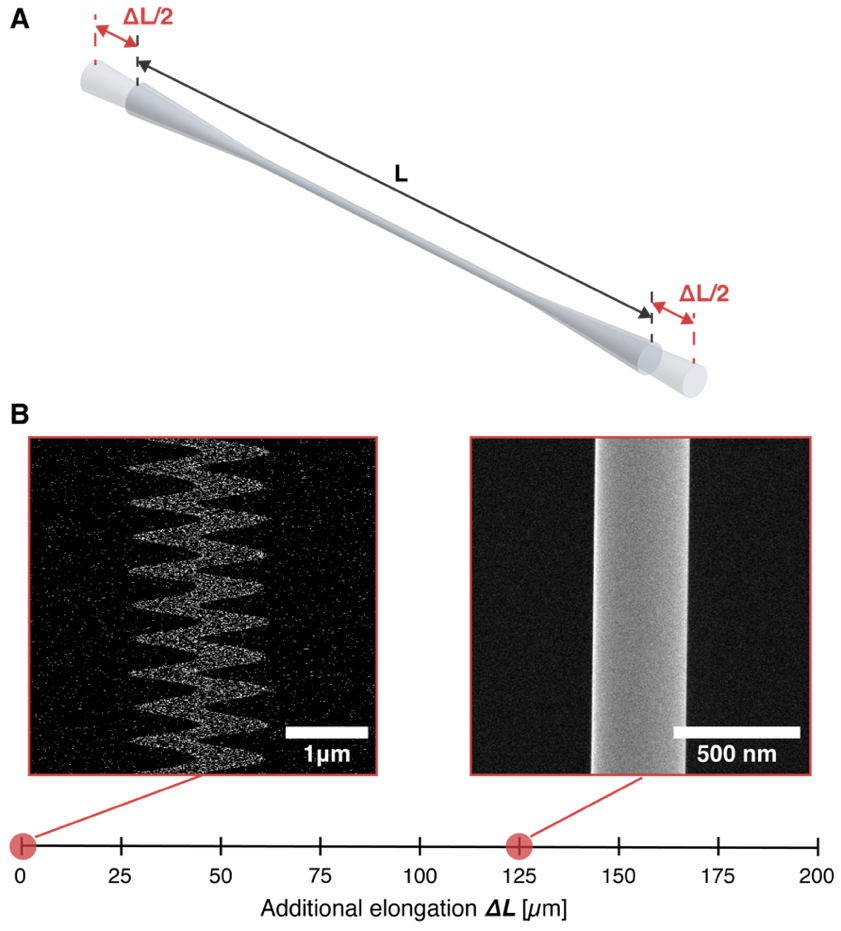


Supplementary Figure S9. Additional elongation. A) Sketch of a TNF of length L at which an elongation of ΔL/2 on each end of the TNF is applied B) SEM micrographs of two d = 360 nm TNFs. On the left, no additional elongation has been applied after the pulling. On the right, a total additional elongation of L = 125 µm has been applied, thus enabling stable imaging of the TNF.

# Plasma Oxygen treatment

## Cleaning of the nanofiber

Accumulation of dust on the nanofiber poses a significant problem, as it leads to a drastic reduction in transmission, often dropping below 10% [[*M. Fujiwara, K. Toubaru, and S. Takeuchi, "Optical transmittance degradation in tapered fibers," Opt. Express 19, 8596-8601 (2011)*](https://opg.optica.org/oe/fulltext.cfm?uri=oe-19-9-8596&id=212829)]. To address this issue, we employed a Plasma Oxygen treatment. This method, commonly used to clean macroscopic flat substrates, is highly effective at removing contaminants at the nanoscale compared to traditional wet cleaning methods, such as solvent cleaning.

**Supplementary Figure S10** showcases the effectiveness of this treatment on a nanofiber: the upper panel shows the effect of light scattering from dust when a laser is directed into a nanofiber. Under these conditions, it becomes nearly impossible to distinguish between the nanostructure and the dust, as both act as scattering centers. The bottom panel displays the result after plasma cleaning, where the nanostructure becomes clearly visible and the nanofiber is completely free of dust.


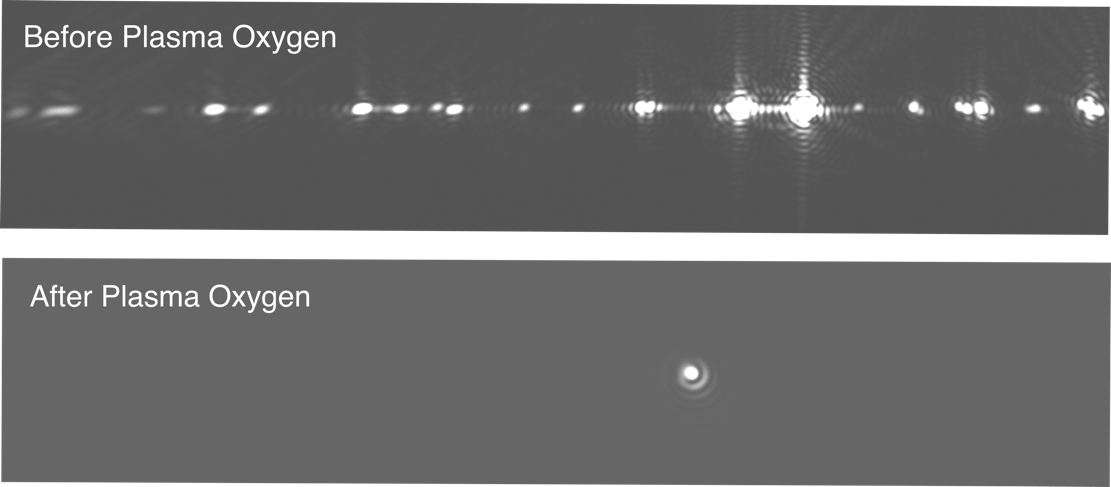


Supplementary Figure S10. Plasma Oxygen cleaning effect. (upper panel) Before Plasma Oxygen treatment light scattering from dust heavily masks the scattering from the fabricated structures when a laser is sent into the nanofiber. (bottom panel) After Plasma Oxygen treatment the dust is removed from the nanofiber and only the scattering from the nanostructure is visible.

## Morphological effects of Plasma Oxygen

The Plasma Oxygen Treatment also leads to ‘purification’ effects on the composition of the EBID nanostructures, reducing the carbon concentration in the nanostructure, thus increasing the overall metal content. In turn, this treatment influences the dimensions of the nanostructures, potentially reducing both the diameter and the height of a few tens of nanometers. The effect has been characterized on a test planar sample (**Supplementary Figure S11**).

**
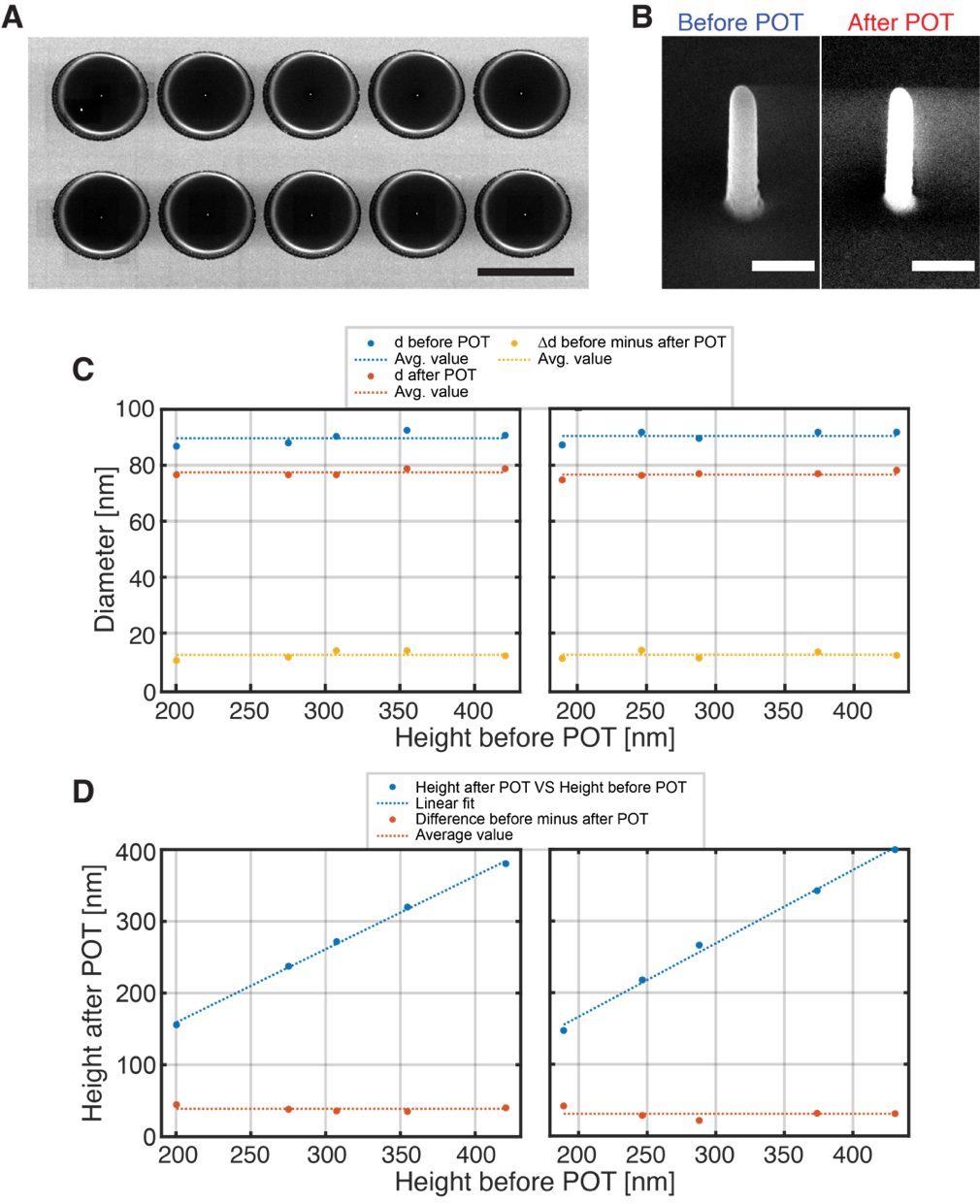
**

Supplementary Figure S11. Plasma Oxygen morphological effect. A) SEM micrograph of the test structures consisting of 3 µm-diameter 1 µm-depth holes milled by FIB milling in a 100 nm-thick gold film deposited on a glass substrate. At the center of each hole, a single nanopillar is grown, increasing the nominal height from 200 to 400 nm, keeping a constant diameter of 100 nm. The scale bar is 3 µm. B) Example SEM micrograph of a single pillar used for SEM inspection before and after the exposure to the plasma oxygen. Scale bars are 200 nm. C) Results of the SEM inspection to determine the diameter reduction after the plasma oxygen exposure on the upper row pillars (left) and the lower row ones (right). D) Results of the SEM inspection to determine the height reduction after the plasma oxygen exposure on the upper row pillars (left) and the lower row ones (right).

# Experimental and numerical supplementary material

**
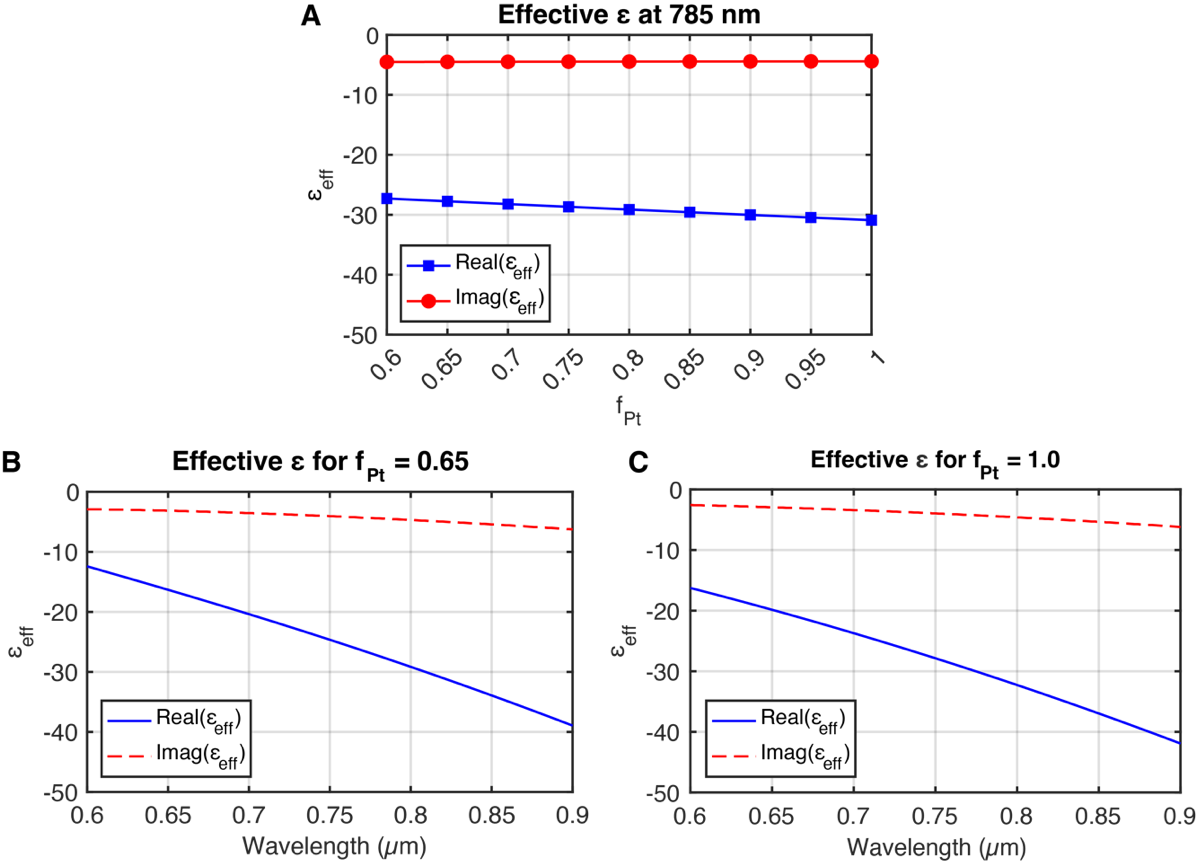
**

Supplementary Figure S12. Effective medium theory calculations. A) Calculated effective dielectric permittivity at a fixed wavelength of λ = 785 nm for f_Pt_ ∈ [0.6, 1.0] at steps of 0.05. B) Calculated effective dielectric permittivity for f_Pt_ = 0.65. C) Calculated effective dielectric permittivity for f_Pt_ = 1.00.


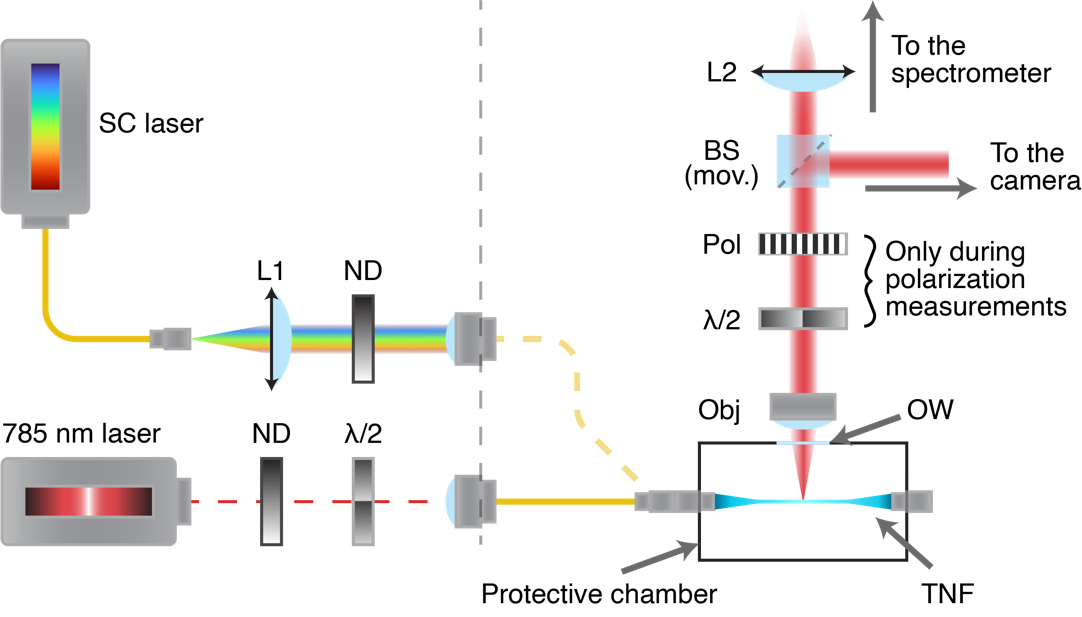


Supplementary Figure S13. Sketch of the measurement setup. Two laser sources can be selectively sent in input inside the TNF: a supercontinuum source for the broadband scattering measurements, and a 785 nm laser for the polarization measurements. The TNF is kept inside an airtight Plexiglas container that prevents dust contamination during the experiments. The container is equipped with an optical window (Thorlabs WG41010R-B) with R < 0.5% in the wavelength range of interest. The signal is collected by an objective lens and sent toward the spectrometer. During the polarization measurement, a half waveplate and a polarizer are inserted in the optical path, as described in the main text. The sample is monitored thanks to an imaging branch enabled by a beam splitter that can be removed during the measurements, to maximize the collected signal.

***
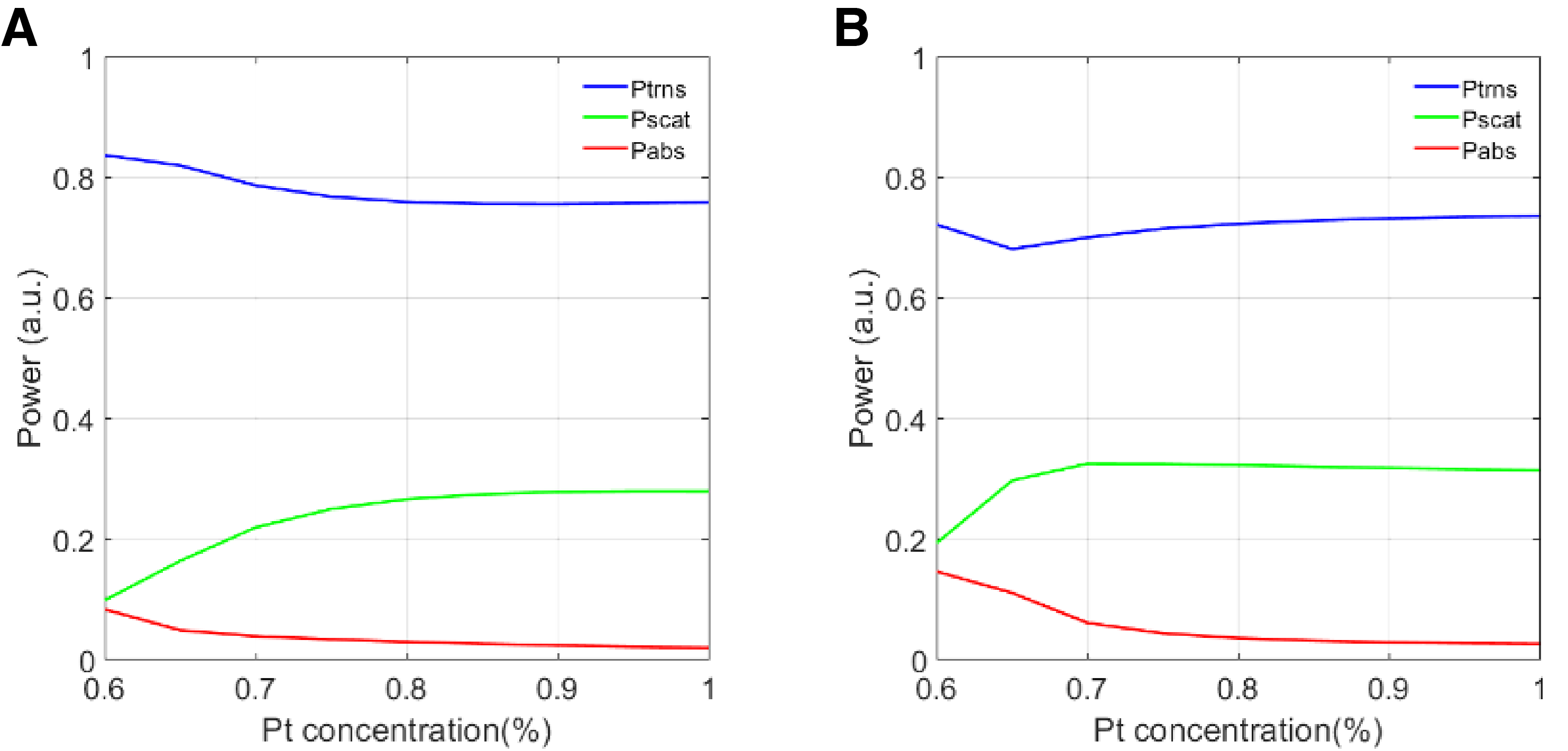
***

***Supplementary Figure S14. Computed transmission, scattering, and absorption. A)*** *Numerical simulations of the relative contributions of transmission, scattering, and absorption, repeated across Pt contents from 60% to 100% for the nanopillar configuration.* ***B)*** *Corresponding results for the nanoantenna configuration.*


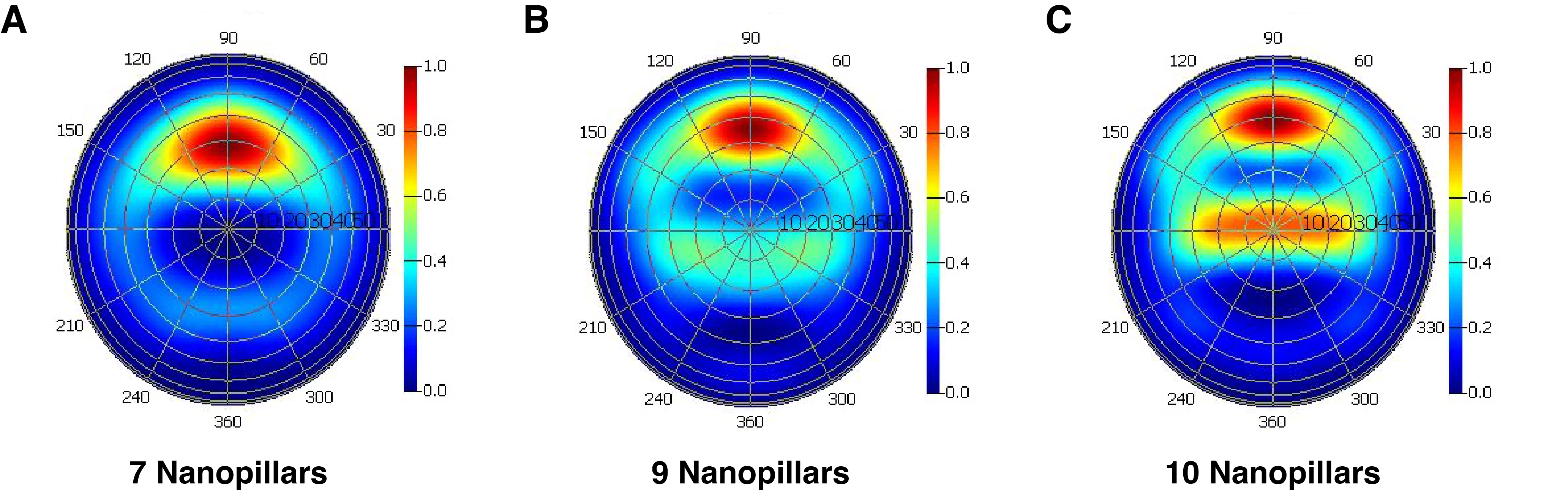


Supplementary Figure S15. Normalized scattered far field intensity (|E|^2^) on a 2π hemisphere enclosing the nanopillars. A-C) Polar maps of the far-field intensity distribution of nano-pillar arrays containing, respectively, 7 nanopillars, 9 nanopillars, and 10 nanopillars.
